# Comparison the impact of IFN-β alone or in combination with vitamin D on critical pathways involved in AML progression

**DOI:** 10.1371/journal.pone.0330865

**Published:** 2025-10-16

**Authors:** Farzad Nasri, Hassan Jalaeikhoo, Maryam Dadmanesh, Nafiseh Behranvand, Reza Falak, Negin Hosseini Rouzbahan, Yalda Babaei, Khodayar Ghorban

**Affiliations:** 1 Infectious Diseases Research Center, Aja University of Medical Sciences, Tehran, Iran; 2 Department of Immunology, Faculty of Medicine, Aja University of Medical Sciences, Tehran, Iran; 3 Department of Immunology, School of Medicine, Iran University of Medical Sciences, Tehran, Iran; 4 Department of Hematology and Oncology, Faculty of Medicine, Aja University of Medical Sciences, Tehran, Iran; 5 Department of Infectious Diseases, Faculty of Medicine, Aja University of Medical Sciences, Tehran, Iran; 6 Department of Laboratory Sciences, School of Paramedicine, Shahid Sadoughi University of Medical Sciences, Yazd, Iran; Faculty of Medicine of Tunis, TUNISIA

## Abstract

**Background:**

Acute myeloid leukemia (AML) is a malignant disorder characterized by the accumulation of immature myeloid cells which can be developed and exacerbated through inflammation. Interferon-β (IFN-β) and vitamin D (Vit D) are known for their immunomodulatory and anti-proliferative effects. Key molecules like IL-1β, Gal-9, β-catenin, and NF-κB play important roles in AML progression. This study examines the effects of IFN-β alone and in combination with Vit D on U937 cell proliferation and the regulation of these key molecules.

**Methods:**

Cell counting kit-8 (CCK-8) was applied to assess the effect of IFN-β and Vit D on U937 cells proliferation. Real-time PCR was carried out to evaluate the impact of IFN-β and Vit D on *IL-1β, IL-10, Gal-9, β-*catenin**, and *NF-κ*B** genes. ELISA was performed to estimate the protein levels of IL-1β and IL-10. Western-blotting was applied to evaluate the NF-κB signaling pathway.

**Results:**

The proliferation of U937 cells reduced significantly in the presence of IFN-β, while it did not show a remarkable change following treatment with Vit D. *IL-1β* gene expression was reduced following either IFN-β or Vit D treatment, while *IL-10* gene expression underwent a gentl increase following treatment with IFN-β, which was in contrast to Vit D treatment. IFN-β in contrast to Vit D decreased *Gal-9* gene expression. *β-*catenin** gene expression increased after IFN-β or Vit D treatment. *NF-κ*B** gene expression reduced following either IFN-β or Vit D treatment, except for the highest concentration of Vit D. At protein level, IFN-β and Vit D treatment leads to a reduction of both IL-1β and IL-10 as well as p-NF-κB.

**Conclusion:**

Our findings suggest that IFN-β effectively inhibits U937 cell proliferation and modulates key inflammatory and immune-related molecules involved in AML pathogenesis. These results highlight IFN-β as a promising agent for targeting critical pathways in AML and suggest a modulatory, but less potent role for Vit D.

## Introduction

Acute myeloid leukemia (AML) is a heterogeneous clonal disorder in which myeloid lineage cells proliferate out of control, which can interfere with the production of normal cells in the bone marrow [1–3]. AML is the most prevalent acute leukemia in adults, approximately 150,000 new cases were diagnosed worldwide, only in 2021 [4]. There has been no change in the AML standard therapy procedures for years, and the 3 + 7 regimen of daunorubicin and cytarabine is still commonly used. However, the high mortality rate of AML confirms insufficient efficacy of the current therapy methods [5,6].

The growing cell biology knowledge has paved the way for development of novel and promising strategies in AML treatment [7–12]. Type I interferon (IFN) was first recognized as an antiviral cytokine [13], which has recently been introduced as a useful therapeutic approach against solid tumors [14], and the role of this cytokine has been confirmed in several blood malignancies, as well [15,16]. The studies show that Type 1 interferon can have both inflammatory and anti-inflammatory effects, in a way that the formation of signal transducer and activator of transcription-1/2 (STAT-1/STAT2) heterodimer increases the expression of inflammatory genes, while homodimerization of each STAT-1 or STAT-3 molecules suppresses their expression [17–19]. One of the most pivotal roles of IFN-β is prohibiting the production of interleukin 1-β (IL1-β), a critical cytokine in expansion of AML cells. Furthermore, some experiments have revealed that IFN-β can increase the production of IL-10 as an anti-inflammatory cytokine through SATAT-3 signaling [20].

Inflammatory cytokines play a critical role in AML development, in a way that the people who are older than 60 years are more susceptible to this leukemia due to expressing a remarkable higher amount of inflammatory cytokines, a phenomenon known as inflammaging, indicating that there might be an association between age, inflammation, and AML incidence [21,22]. Among all; IL-1β is one of the most important inflammatory cytokines, which has been proven to play a significant role in promoting AML. Findings indicate the fundamental impact of IL-1β in the myeloid progenitor proliferation, in a way that P38MAPK inhibition, which plays a role in IL-1β signal transmission, leads to the blockade of AML blast cells proliferation [23–26].

Various aspects of vitamin D (Vit D) have been investigated, which shows its importance in the body’s physiologic and immune system reactions [27,28]. According to reports Vit D may play a role in the transformation of monocytes into mature macrophages. Today’s studies show that this vitamin modulates inflammation through decreasing the production of inflammatory cytokines and shifting the immune response from Th-1 to Th-2. In addition, this molecule has the potential to enhance the suppressive activity of regulatory cells [29,30].

In 2019, Xuan Feng, et al. showed that Vit D plays a crucial role in IFN-β signal transmission, improving its therapeutic performance in the treatment of multiple sclerosis, by increasing the IFN-β signal transmission through STAT-1, which, as mentioned earlier, can play an important role in the suppressing the production of inflammatory cytokines such as IL-1 and increasing the production of anti-inflammatory cytokines such as IL-10 [31,32]. The addition of Vit D to IFN-β has reversed some of the effects of IFN-β, on its own. IFN-β and Vit D combination therapy decreased the production of Th-1 and Th-17 inflammatory cytokines, but increased serum levels of Th-2 anti-inflammatory cytokines (IL-4, IL-5, and IL-10). Vit D modulates IFN-β responses in both lymphocytes and monocytes [19,33,34].

In this study, we examined weather application of IFN-β or Vit D alone or in combination in acute myeloid leukemia cells can cause an inflammatory or anti-inflammatory response.

## Materials and methods

### Cell culture

U937 cells were purchased from the Stem Cell Technology Research Center (Tehran, Iran). Cells were propagated in RPMI-1640, supplemented with 10% fetal bovine serum (FBS) and 1% penicillin-streptomycin and maintained at 37 °C in a humidified CO_2_ incubator containing 5% CO_2_. During all experiments, cells were at their logarithmic growth stage and primed with determined concentrations of IFN-β (100, 1000, 10000 ng/ml) (Ziferon, Tehran, Iran) and Vit D (50, 500, 5000 ng/ml) (Dithrecol, Tehran, Iran). Co-treatment (Co) groups were simultaneous treatment of the cells with both IFN-β and Vit D including Co-1 (100 ng/ml IFN-β + 50 ng/ml Vit D), Co-2 (1000 ng/ml IFN-β + 500 ng/ml Vit D), and Co-3 (10000 ng/ml IFN-β + 5000 ng/ml Vit D).

### Proliferation assay

The proliferation of U937 cells in the presence of IFN-β and Vit-D was analyzed using cell-counting Kit-8 (CCK-8) (BioLegend, San Diego, CA, USA). In details, 20 × 10^3^ U937 cells were seeded per well in a 96-well plate and treated with different concentrations of IFN-β and Vit D, ranging from 5 ng/ml to 50 µg/ml with a ratio of ten times, for 24 h. After incubation time, 10 µl of CCK-8 solution was added to each well and incubated for three hours. In continue, the production of the water-soluble formazan dye, proportional to viable cell numbers, was measured with a microplate reader at 460 nm.

### Real‑time PCR

Real-time PCR was performed to evaluate the effect of IFN-β and Vit D on expression level of multiple target genes including *IL-1β, IL-10, Galectin-9 (Gal-9), β-*catenin**, and *NF-κ*B**. Briefly, 3 × 10^6^ U937 cells were seeded per well in 6-well plates and primed with 100 ng/ml of lipopolysaccharide from Escherichia coli O111:B4 (LPS, L2630, Sigma-Aldrich), and different concentrations of IFN-β and Vit D. After 24 hours, total RNA was extracted using TRIzol® reagent (DNA biotech, Tehran, Iran) through the guanidinium thiocyanate-phenol-chloroform extraction method. The quality of the extracted RNA was assessed with NanoDrop 2000c spectrophotometer and 1 µg/ml of total RNA was used for c-DNA synthesis based on Easy cDNA Synthesis Kit protocol (Parstous biotechnology, Mashhad, Iran). Real-time PCR was performed as stated in the instructions using the 2x SYBR-Green master mix (Biofact, Daejeon, South Korea). Table 1 shows the sequences of the applied primers. GAPDH was considered as the housekeeping gene and the 2^−(ΔΔCT)^ value was calculated to determine the relative expression of the genes.

**Table 1:** Details of the primers applied in real-time PCR.

Target gen name	Forward and reverse primer sequence	Tm
*GAPDH*	F- GCACCGTCAAGGCTGAGAAC R- TGGTGAAGACGCCAGTGGA	58°C
*NF-κB*	F- TCTTCCCTTTGACTGTGTCCT R- TTCAAACACAGGACAGGCTC	60°C
*Gal-9*	F-GATGAGAATGCTGTGGTCCG R- GAAGCCGCCTATGTCTGCA	58°C
*IL-1β*	F- AGCTTGGTGATGTCTGGTCC R- ACGCAGGACAGGTACAGATT	58°C
*B-catenin*	F-CGGGCTCATTTTGGAACAGAT R-GGCAGACTGTCTCGGTCAA	60°C
*IL-10*	F-TCTCCGAGATGCCTTCAGCAGA R-TCAGACAAGGCTTGGCAACCCA	60°C

### ELISA

IL-1β and IL-10 protein was estimated by ELISA method. In brief, 1.5 × 10^6^ U937 cells were seeded per well in 24-well plates and treated with 100 ng/ml of LPS and different concentrations of IFN-β and Vit D for 24 h. After incubation time, cells were lysed with radioimmunoprecipitation assay buffer (RIPA; Santa Cruz Biotechnology, Dallas, Texas, USA) and the cell lysates were collected to estimate the IL-1β and IL-10 protein level. ELISA procedure was performed according to Human IL-1β ELISA development kit (MABTECH, Stockholm, Sweden) and Human IL-10 ELISA kit (BioLegend, San Diego, CA, USA) instructions.

### Western blot analysis

Western blotting was conducted to determine the effect of IFN-β and Vit D on the total and phosphorylated forms of NF-κB. In details, 5 × 10^6^ cells were seeded per well in 6-well plates and stimulated with 100 ng/ml of LPS, 100000 ng/ml of IFN-β and 5000 ng/ml Vit D. Cells were harvested, washed with cold phosphate buffered saline (PBS), and lysed with RIPA solution containing protease and phosphatase inhibitor cocktail. Protein concentration of the whole cell lysates was measured by bicinchoninic acid (BCA) kit (DNA biotech, Tehran, Iran), and equal amount of protein (60 μg) were loaded onto a 12% SDS-PAGE gel. The resolved proteins were electro-transferred to the polyvinylidene difluoride (PVDF) membrane. The membrane was blocked with 5% skimmed milk in PBS containing 0.2% tween 20 (PBST), for 2 h at room temperature. The membrane was then incubated with 1:200 dilution of mouse anti-human NF-κB p65 (Santa Cruz Biotechnology, Dallas, Texas), 1:200 dilution mouse anti-human p-NF-κB p65 (Santa Cruz Biotechnology, Dallas, Texas), and 1:1000 dilution of β actin (Abcam, Cambridge, UK), diluted in 2% skim milk/PBST, and incubated at 4 °C overnight, followed by three washes (each 5 min) in PBST. In continue, the membrane was incubated with appropriate dilutions of secondary antibodies for 1 h at room temperature, followed by 4 washes with PBST. Visualization of protein bands was carried out using enhanced chemiluminescence reagent (Intron Biotechnology, Seongnam-Si, South Korea).

### Statistical analysis

Data analysis was conducted using Graph Pad Prism software 9 (Graph Pad Prism Software, San Diego, CA, USA). One-way ANOVA followed by a Bonferroni-Dunn post-hoc test was done to compare more than two independent groups. The results were expressed as the mean ± standard deviation (SD). Data distribution was examined using the Kolmogorov-Smirnov test and variables that weren’t normal were analyzed by non-parametric tests. To analyze western blot results, nonparametric tests were performed. Kruskal–Wallis, Dunn test was performed to compare different independent groups. Moreover, Benjamini–Hochberg was done to control the False Discovery Rate (FDR) in multiple testing experiments. The data are presented as the median ± interquartile range (IQR). P < 0.05 was considered as a statistically significant difference between groups.

## Results

### The effect of IFN-β and Vit D on the U937 cells viability

**Fig 1:**
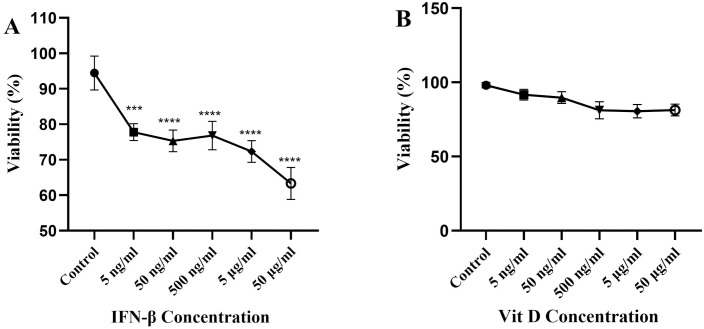
The effect of IFN-β and Vit D on the viability of U937 cells. U937 cells were treated with a wide range of IFN-β and Vit D concentrations and incubated for 24 **h.** Different concentrations of IFN-β induced cell toxicity compared to the control which was statistically significant, but the cytotoxicity of none of the concentrations was more than 50% **(A)**. Different concentrations of Vit D did not have any significant proliferative or cytotoxic effects on U937 cells **(B)**. Data represents the mean (± SD) of fold changes from two independent experiments, each performed in triplicate using One-way ANOVA, Bonferroni test. (****p < 0.0001).

The viability of U937 cells following treatment with a wide range of IFN-β concentrations, decreased significantly in comparison to the control. It is noteworthy to mention that none of the applied concentrations caused more than 50% cytotoxicity; therefore we chose 100, 1000, 10000 ng/ml concentrations for the following experiments (Fig 1A) (*p* < 0.0001). We also primed U937 cells with different concentrations of Vit D and neither significant proliferation nor cytotoxicity was seen compared to the control, so 50, 500, 5000 ng/ml concentrations were selected for the following experiments (Fig 1B).

### The effect of IFN-β and Vit D on the *IL-1β* gene expression

**Fig 2:**
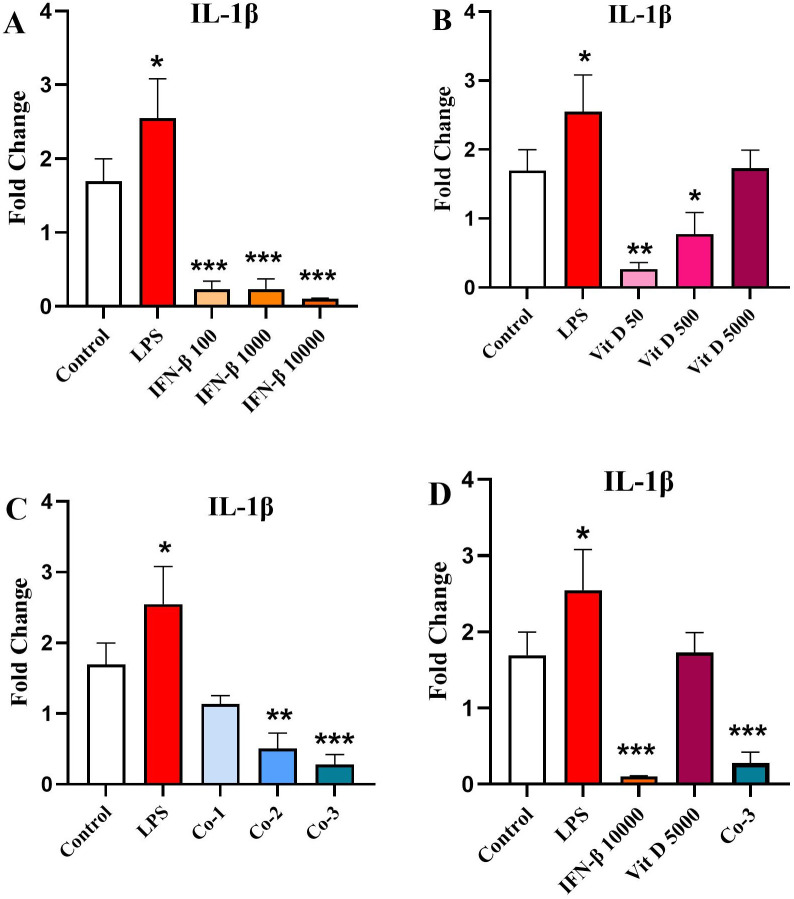
The effect of IFN-β and Vit D on the IL-1β expression. U937 cells were treated with different concentrations of IFN-β and Vit D alone or in combination. IFN-β reduced IL-1β expression compared to the control, significantly **(A)**. Also, Vit D induced a significant reduction in the IL-1β expression compared to the control, especially in lower concentrations **(B)**. In the co-treatment groups, a significant dose-dependent decrease was seen in the IL-1β expression **(C)**. In the highest concentration groups, IFN-β alone and the co-treatment were able to reduce IL-1β expression compared to the control, significantly **(D)**. Data represents the mean (± SD) of fold changes from two independent experiments, each performed in triplicate using One-way ANOVA, Bonferroni test. (*p < 0.05, **p < 0.01, ***p < 0.001).

IFN-β was capable to significantly reduce *IL-1β* expression in comparison to the control (*p* < 0.001). However, there was no significant difference among various concentrations (Fig 2A). Vit D decreased *IL-1β* expression compared to the control in a dose-dependent manner, in a way that in the lower concentrations it had more significant effects (*p* < 0.01 and *p* < 0.05 for 50 and 500 ng/ml, respectively) (Fig 2B). In the co-treatment groups, there was a steady downward pattern in the *IL-1β* expression in comparison with the control, which was statistically significant in the second and third groups (*p* < 0.01 and 0.001, respectively) (Fig 2C). We also compared the highest concentration of each group and showed that there was a significant decrease in the *IL-1β* expression in the IFN-β and co-treatment groups compared to the control (*p* < 0.001) (Fig 2D).

### The effect of IFN-β and Vit D on the *IL-10* gene expression

**Fig 3:**
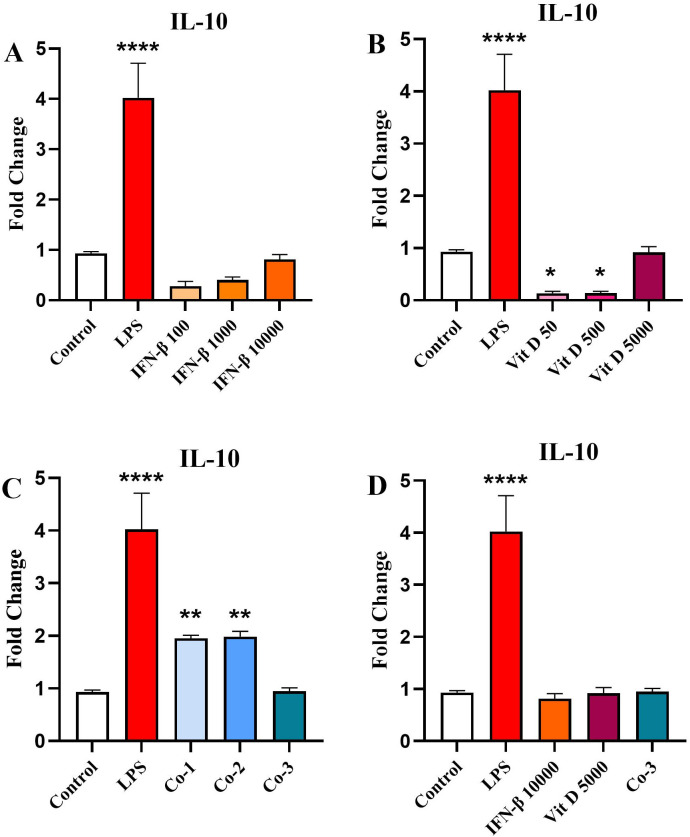
The effect of IFN-β and Vit D on the IL-10 gene expression. U937 cells were treated with different concentrations of IFN-β and Vit D alone or in combination. IFN-β increased IL-10 expression in a dose-dependent manner compared to the control, but not significantly **(A)**. Vit D induced a significant reduction in the IL-10 expression compared to the control especially in lower concentrations **(B)**. In the co-treatment groups, a significant increase was seen in the IL-10 expression in the first and second groups **(C)**. In the highest concentration groups, there was no difference among three groups in IL-10 expression compared to the control **(D)**. Data represents the mean (± SD) of fold changes from two independent experiments, each performed in triplicate using One-way ANOVA, Bonferroni test. (*p < 0.05, **p < 0.01, ****p < 0.0001).

The results revealed that IFN-β could increase *IL-10* expression in a steadily dose-dependent manner, but it was not significant compared to the control (Fig 3A). In contrast, Vit D significantly reduced *IL-10* expression compared to the control, particularly at low concentrations (*p* < 0.05) (Fig 3 B). In the co-treatment groups, a significant increase in the *IL-10* expression was seen in the first and second groups (Fig 3C) (*p* < 0.01). When we compared the highest concentration groups with the control, there was no significant difference (Fig 3D).

### The effect of IFN-β and Vit D on the *Gal-9* gene expression

**Fig 4:**
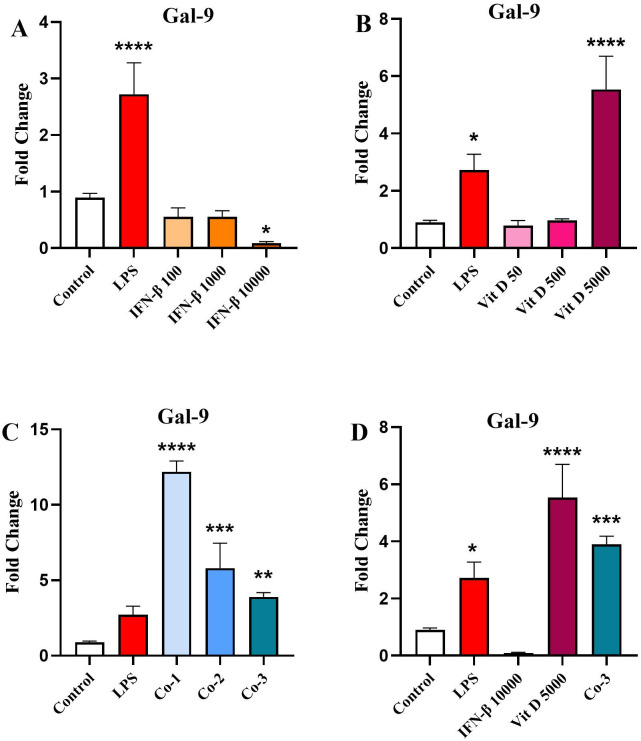
The effect of IFN-β and Vit D on Gal-9 expression. U937 cells were treated with different concentration of IFN-B and Vit D alone or in combination. IFN-β reduced Gal-9 expression compared to the control, especially in the highest concentration **(A)**. Vit D induced a significant increase in the Gal-9 expression compared to the control only in the highest concentration **(B)**. In the co-treatment groups, a significant increase was seen in the Gal-9 expression in a downward pattern **(C)**. In the highest concentration groups, IFN-β reduced Gal-9 expression, while Vit D and Co-3 significantly increased Gal-9 expression compared to the control **(D)**. Data represents the mean (±SD) of fold changes from two independent experiments, each performed in triplicate using One-way ANOVA, Bonferroni test. (*p < 0.05, **p < 0.01, ***p < 0.001, ****p < 0.0001).

INF-β could reduce the expression of *Gal-9* in comparison to the control. However, it was statistically significant only for the highest concentration (Fig 4A). On the opposite, Vit D had no effect on *Gal-9* expression in two lower concentrations, but the highest concentration significantly augmented *Gal-9* expression compared to the control (Fig 4B) (*p* < 0.0001). In the co-treatment groups, *Gal-9* expression was significantly increased in all groups compared to the control which was in a downward dose-dependent pattern (Fig 4C) (*p* < 0.0001, *p* < 0.001, and *p* < 0.01 for Co-1, Co-2 and Co-3, respectively). Finally, we compared the highest concentration of each group, and it was seen that IFN-β reduced *Gal-9* expression in comparison with the control but not significantly. Vit D and Co-3 significantly increased *Gal-9* compared to the control (Fig 4D) (p < 0.0001, p < 0.001, respectively).

### The effect of IFN-β and Vit D on the *β-catenin* gene expression

**Fig 5:**
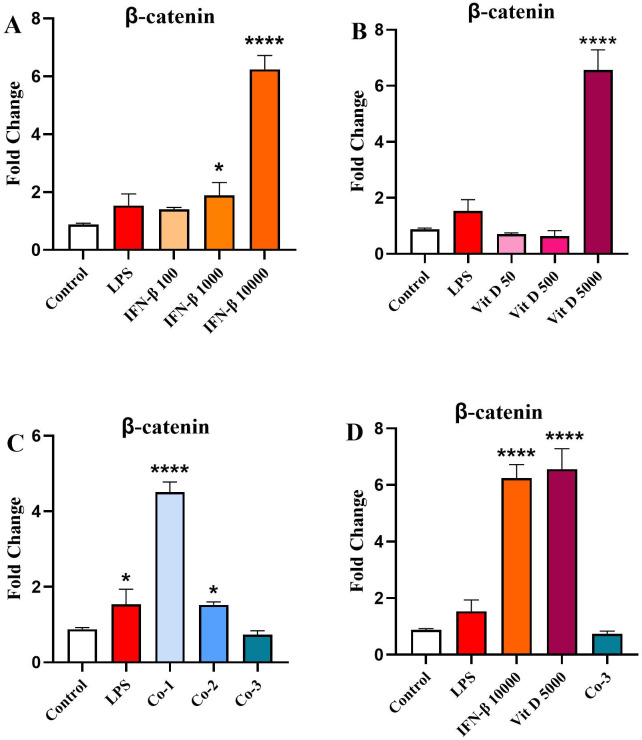
The effect of IFN-β and Vit D on the β-catenin gene expression. U937 cells were treated with different concentrations of IFN-β and Vit D alone or in combination. IFN-β increased β-catenin expression in a dose-dependent manner compared to the control **(A)**. Vit D induced a significant increase in the β-catenin expression in comparison with the control only in the highest concentration **(B)**. In the co-treatment groups, there was a significant increase in the β-catenin expression in a reverse dose-dependent pattern **(C)**. In the highest concentration groups, IFN-β and Vit D increased β-catenin expression significantly compared to the control, but Co-3 caused a minor reduction **(D)**. Data represents the mean (± SD) of fold changes from two independent experiments, each performed in triplicate using One-way ANOVA, Bonferroni test. (*p < 0.05, ****p < 0.0001).

IFN-β could increase *β-catenin* expression in a dose-dependent manner in comparison to the control, which was only significant for 1000 ng/ml (*p* < 0.05) and 10000 ng/ml (*p* < 0.0001) (Fig 5A). Conversely, only the highest concentration of Vit D was able to increase *β-catenin* expression significantly compared to the control (*p* < 0.0001) (Fig 5B). In the co-treatment groups, the results revealed that *β-catenin* expression significantly increased in a reverse dose-dependent manner compared to the control (Fig 5C) (*p* < 0.0001 and *p* < 0.05 for Co-1 and Co-2, respectively). In addition, comparison among highest concentrations showed that IFN-β and Vit D both could augment *β-catenin* expression compared to the control, which was statistically significant (*p* < 0.0001), but Co-3 just induced an imperceptible reduction in comparison with the control (Fig 5D).

### The effect of IFN-β and Vit D on the *NF-κB* gene expression

**Fig 6:**
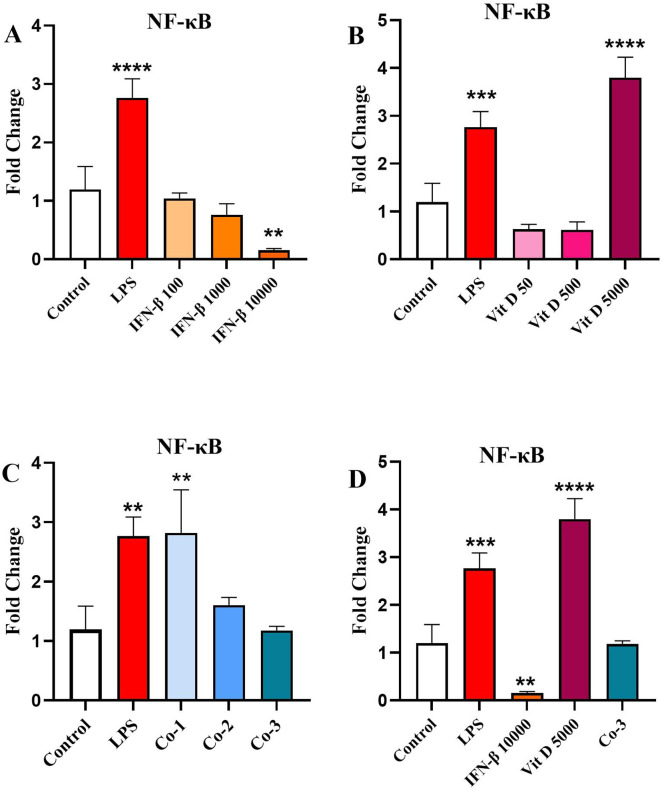
The effect of IFN-β and Vit D on the NF-κB gene expression. U937 cells were treated with different concentrations of IFN-β and Vit D alone or in combination. IFN-β reduced NF-κB expression compared to the control, in a dose-dependent manner **(A)**. Two lower concentrations of Vit D induced a reduction in the NF-κB expression compared to the control, while the highest concentration increased it significantly **(B)**. In the co-treatment groups, a dose-dependent decrease pattern among different concentration was seen in the NF-κB expression, but generally it was increased compared to the control **(C)**. In the highest concentration groups, IFN-β alone was able to significantly reduce NF-κB expression, but Vit D increased it and Co-3 group did not make any differences compared to the control **(D)**. Data represents the mean (± SD) of fold changes from two independent experiments, each performed in triplicate using One-way ANOVA, Bonferroni test. (**p < 0.01, ***p < 0.001, ****p < 0.0001).

IFN-β was capable to decrease *NF-κ*B** expression in a dose-dependent manner compared to the control and it was only significant for the highest concentration (Fig 6A) (*p* < 0.01). *NF-κ*B** expression was reduced by the two lower concentrations of Vit D, but it was not significant. Although, the highest concentration significantly augmented *NF-κ*B** expression compared to the control (Fig 6B) (*p* < 0.0001). In the co-treatment groups, although there was a steady downward dose-dependent pattern among different concentrations, *NF-κ*B** expression increased compared to the control (Fig 6C) (*p* < 0.01). When we compared the highest concentration groups, we saw that IFN-β significantly reduced *NF-κ*B** expression, while Vit D increased it, but the Co-3 group showed no difference with the control (Fig 6D).

### The effect of IFN-β and Vit D on the IL-1β protein

**Fig 7:**
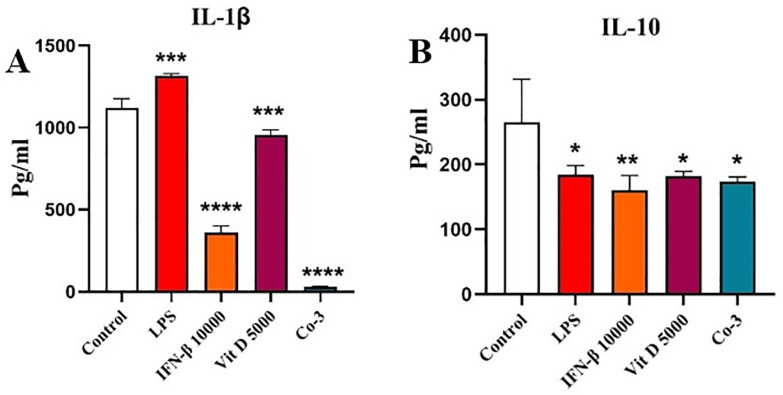
The effect of IFN-β and Vit D on the IL-1β and IL-10 protein production. U937 cells were treated with specific concentrations of IFN-β and Vit D alone or in combination. IFN-β and Vit D alone or together decreased IL-1β and IL-10 protein level, significantly in comparison with the control **(A, B)**. Data represents the mean (± SD) of fold changes from two independent experiments, each performed in triplicate using One-way ANOVA, Bonferroni test. (*p < 0.05, **p < 0.01, ***p < 0.001, ****p < 0.0001).

IFN-β and Vit D both significantly decreased IL-1β protein level, in comparison with the control, and notably IFN-β had a stronger effect than Vit D (*p* < 0.0001 and *p* < 0.001 for IFN-β and Vit D, respectively). In the co-treatment groups, their impact seemed to be reinforced and IL-1β production was significantly reduced compared to the control (Fig 7A) (*p* < 0.0001).

### The effect of IFN-β and Vit D on the IL-10 protein

We found that IFN-β and Vit D alone or in combination together could decrease IL-10 protein level compared to the control, which was statistically significant (Fig 7B) (*p* < 0.01 and 0.05).

### The effect of IFN-β and Vit D on the NF-κB and p-NF-κB protein

**Fig 8:**
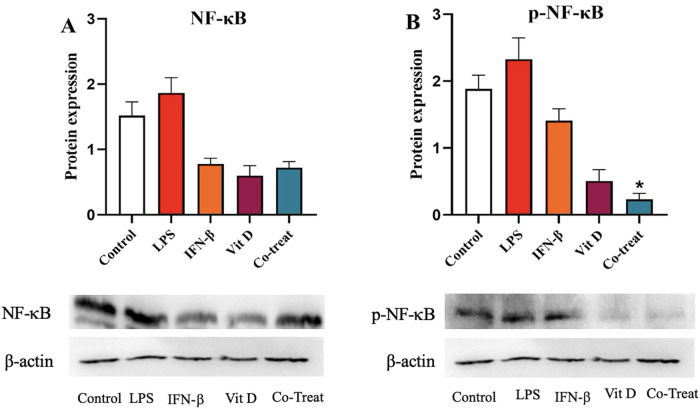
The effect of IFN-β and Vit D on the NF-κB and p-NF-κB protein. U937 cells were treated with specific concentrations of IFN-β and Vit D alone and in combination. NF-κB protein expression was reduced in all groups compared to the control, but not significantly **(A)**. IFN-β, Vit D, and the co-treatment all decrease p-NF-κB protein expression in comparison with the control, but it was only significant for the co-treatment group **(B)**. Data represent the median (± IQR) of normalized protein expression with β-actin from experiments, which were done in a triplicate manner. Statistical analysis was performed on protein expression, using Kruskal–Wallis, Dunn test, and Benjamini–Hochberg. Error bars indicate IQR. (*p < 0.05).

All of the treatments including IFN-β, Vit D, and the co-treatment decreased NF-κB protein in comparison to the control, but it was not statistically significant. Besides, there was no remarkable difference among different groups (Fig 8A). We also evaluated the effect of the treatments on phosphorylation of NF-κB and found that, all groups similarly had lower amount of p-NF-κB compared to the control, although it was only significant for the co-treatment group (Fig 8B) (*p* < 0.05).

## Discussion

AML is one the deadliest malignancies, which causes death of a large population worldwide. Some studies have shown the impact of inflammation on AML exacerbation and have introduced NF-κB and IL-1β as two key inflammatory factors responsible for AML cells proliferation and therapy-resistant [23].

IFN-β plays a pivotal role in anti-viral defenses. Also, it effectively prohibits cancer cell growth, so its administration in some solid tumors have been in the center of attention, recently. IFN-β is capable of inducing both inflammatory and anti-inflammatory signaling pathways. Meanwhile, Vit D could affect IFN-β signaling pathway and reduce the production of inflammatory cytokines [33].

In this study, we aimed to evaluate the effect of IFN-β, alone or in combination with Vit D, on proliferation of the U937 cells as an AML cell line and expression of some inflammatory factors as well. First, we assessed the effect of a wide range of IFN-β and Vit D concentrations on U937 cells to examine whether they are cytotoxic or not. IFN-β decreased U937 cells viability significantly, while Vit D did not induce significant cytotoxicity. Consistent with our findings, Holiseck et al. study indicated that type I IFN has anti-cancer impact. Their findings revealed that despite the release of type I interferons by cancer cells, the function of these cytokines is hindered in AML patients [15]. They also showed that type I IFNs independently are related to better relapse-free survival and overall survival of the patients. Moreover, Musella et al., revealed that type I IFNs not only inhibit tumor cell proliferation but also enhance anti-leukemic immunity through epigenetic modulation and reprogramming of the immune microenvironment [35]. However, other studies in colorectal cancer, breast cancer, and glioblastoma indicated that type I IFNs signaling is associated with poor disease outcome [36–39]. The discrepancy, which appears to be present, could be due to the unique implications of potent/acute and indolent/chronic type I IFNs signaling, malfunction of type I IFNs, as well as the overall immunological environment in the cancer.

Studies have shown Vit D modifies the phenotype of U937 cell line without changing cell proliferation [40]. According to another study, the expression of Vit D receptor on the myelomonocytic lineage, like U937 cells, may protect against programmed cell death [41]. In previous studies most of the blasts isolated from AML patients did not respond to Vit D or its analogs [42,43] Moreover, Wimalawansa et al., showed the immunomodulatory effects of Vit D across hematologic malignancies, suggesting that it could serve as a supportive agent for modulating inflammation without strong cytotoxic effects [44].

Afterwards, we assessed the effect of IFN-β and Vit D on both gene expression and protein level of IL-1β, which is a critical inflammatory cytokine that contributes to the proliferation and resistance of AML cells to the therapy. We showed that, although there was no significant difference among different concentrations, IL-1β expression decreased significantly compared to the control. Besides, Vit D was not effective at the highest concentration, but it reduced IL-1β expression in two other lower concentrations. In the co-treatment groups, a dose-dependent reduction in IL-1β expression was observed. At the protein level, both IFN-β and Vit D were able to reduce the levels of IL-1β protein, but IFN-β had a stronger effect. Similarly, in the co-treatment groups, a sever reduction in the levels of IL-1β protein was seen, which may indicate a synergistic effect between IFN-β and Vit D.

Our findings support the promising effects of Vit D therapy for AML patients. Ge et al. revealed that Vit D suppresses LPS-induced IL-1β production through regulation of hypoxia-inducible factor-1α (HIF-1α) signaling pathway [45]. It has been observed that IFN-α and IFN-β suppress the transcription and translation of IL-1β in different cell types [46–49].

In the next step, we examined the effect of IFN-β and Vit D on the IL-10 gene expression and its protein level as an anti-inflammatory cytokine. We figured out that IFN-β modestly increased IL-10 expression, while Vit D reduced its expression, especially in lower concentrations. Co-treatment led to a significant upregulation of IL-10 expression, implying that combined therapy might shift the immune response towards an anti-inflammatory state. This is consistent with findings by Feng et al., who showed that Vit D enhances IFN-β responses by promoting STAT-1 activation and thereby modulating IL-10 production [50]. At the protein level, all groups could decrease IL-10 protein level compared to the control, but there was not a significant difference among them.

Despite a significant increase in IL-10 and NF- κB transcripts, the corresponding protein levels declined. This apparent disparity could be due to post-transcriptional regulatory mechanisms, such as microRNA-mediated translational repression or RNA-binding proteins that affect mRNA stability and translation. RNA-binding proteins such as TTP/ZFP36 bind to AREs in cytokine mRNAs and destabilizing them [51]Furthermore, Vitamin D through its receptor (VDR) can promote proteasomal degradation of NF‑κB proteins such as p65, p105, and p100, without altering their transcription [52].

Moreover, the NF-κB signaling network has feedback mechanisms and protein-level regulation that can reduce protein accumulation even when mRNA levels are high [53].

Gal-9 is another vital element in AML exacerbation through constructing an autocrine loop with its receptor (T cell immunoglobulin and mucin domain-containing protein 3 (TIM-3) expressed on the surface of AML cells, which induces fundamental signaling pathways leading to AML cell proliferation [54,55]. We showed that IFN-β could decrease Gal-9 expression, while Vit D increased its expression. It’s worth mentioning that the highest concentration was the most effective for each treatment. In the co-treatment groups, Gal-9 expression was augmented compared to the control, but a downward pattern was seen among different concentrations.

Our recent study showed that Gal-9 induces IL-1β production in U937 cell line [56]. Accordingly, the inhibition of Gal-9 may be responsible for the effect of IFN-β on reducing IL-1β levels. However, more detailed studies on other AML cell lines and leukemia stem cells (LSCs) should be conducted to investigate the effects of IFN-β on other cell death pathways [57].

Finally, considering the point that β-catenin and NF-κB are the two fundamental signaling pathways that are provoked through TIM-3/Gal-9 autocrine loop, we evaluated the effect of IFN-β and Vit D on β-catenin and NF-κB expression. Our assessment also included the impact of IFN-β and Vit D on NF-κB phosphorylation. We found that IFN-β increased β-catenin expression in a dose-dependent manner, while Vit D was only able to increase β-catenin expression at the highest concentration. On the other hand, the co-treatment group completely reversed IFN-β pattern and reduced β-catenin expression in a dose-dependent manner.

Tang et al., showed that Vit D not only can increase β-catenin transcription but also provokes its nuclear translocation, under oxidative stress in human melanocytes [58]. Moreover, Pendás-Franco et al., indicated that Vit D in human colon cancer cells is capable of repressing Wnt antagonist DICKKOPF-4 (DKK-4) gene, which is an extracellular inhibitor of Wnt/β-catenin pathway [59]. In contrast, Aguilera et al., showed that Vit D can trigger DKK-1 gene in human colon tumor cells Also, P Kaler demonstrated that Vit D can inhibit STAT-1 activation through reducing IL-1β production in THP-1 cells, which leads to disruption of Wnt/ β-catenin signaling in tumor cells [60]. The effects of Vit D appear to be context-dependent, capable of both activating and inhibiting β-catenin signaling, depending on the cellular environment and concentration. The co-treatment results suggest that Vit D may exert a dominant inhibitory effect on β-catenin expression, potentially overriding the stimulatory influence of IFN-β.

We also revealed that IFN-β was capable to decrease NF-κB expression in a dose-dependent manner. Conversely, Vit D increased NF-κB expression only at the highest concentration. There was a steady decline in NF-κB expression among different concentrations in the co-treatment group. The results of western blotting showed that, there was no noticeable difference among various treatments in the total form of NF-κB. In opposite, the phosphorylated form of NF-κB (p-NF-κB) was significantly decreased in the co-treatment group, which could be another sign of synergistic effect of IFN-β and Vit D.

Chen et al., demonstrated that Vit D can prohibit canonical activation of NF-κB through direct physical interaction of its receptor with IκB kinase β (IKKβ) [52]. Other studies revealed that Vit D can reduce NF-κB activation and its nucleus translocation through promoting IκBα as an inhibitor [61,62].

## Conclusion

In conclusion, these results support IFN-β as a promising therapeutic candidate in targeting inflammatory pathways in AML and suggest that Vit D, although less potent, could play a modulatory role in combination strategies. Further studies are required to elucidate the precise molecular mechanisms and evaluate the clinical relevance of these findings in AML treatment.

## Supporting information

S1 File This file includes original gel duct pictures for western blotiing which are NF-κB, pNF-κB and β-actin. (PDF)

S2 File This is raw data of viability data. (XML)

S3 File This is raw data of IL-1β real-time PCR. (XML)

S4 File This is raw data of IL-10 real-time PCR. (XML)

S5 File This is raw data of Gal-9 real-time PCR. (XML)

S6 File This is raw data of β-catenin real-time PCR. (XML)

S7 File This is raw data of NF-κB real-time PCR. (XML)

S8 File This is raw data of IL-1β ELISA. (XML)

S9 File This is raw data of IL-10 ELISA. (XML)

S10 File This is raw data of NF-κB and p-NF-κB western-blotting. (XML)
