# Supplementary figures and images for: Comparison the impact of IFN-β alone or in combination with vitamin D on critical pathways involved in AML progression
Source: PLoS One. 2025 Oct 16;20(10):e0330865. doi: 10.1371/journal.pone.0330865 (PMC12530539; doi:10.1371/journal.pone.0330865)

NF-KB

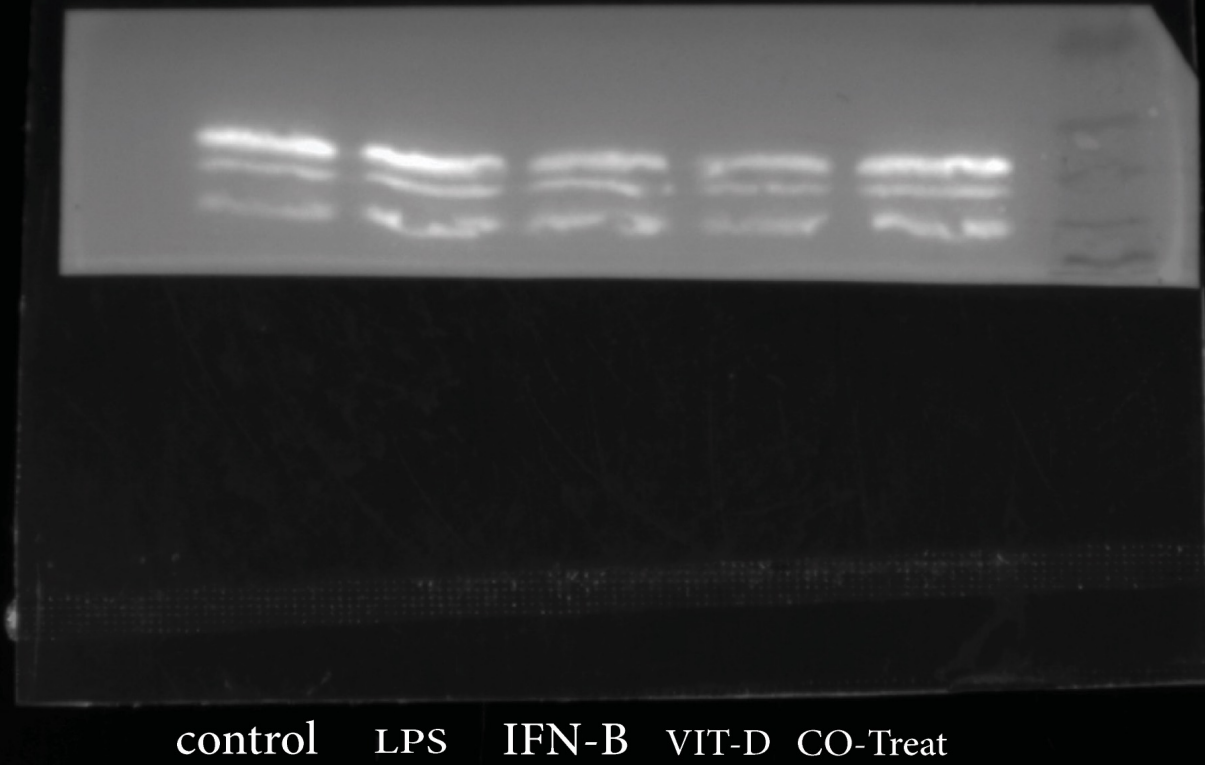

p-NF-KB

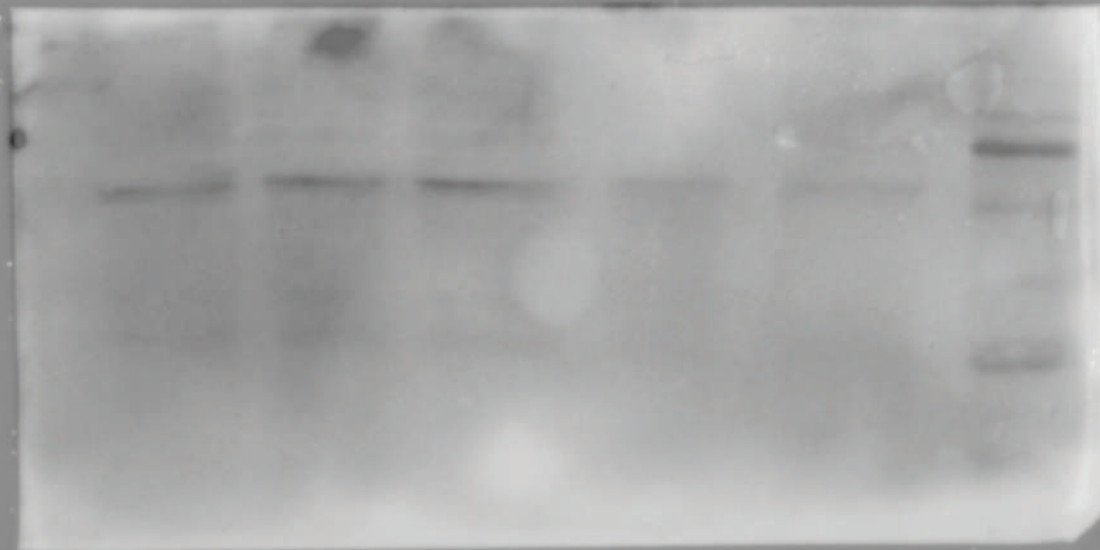

control    LPS    IFN-B    VIT-D    CO-Treat

Actin

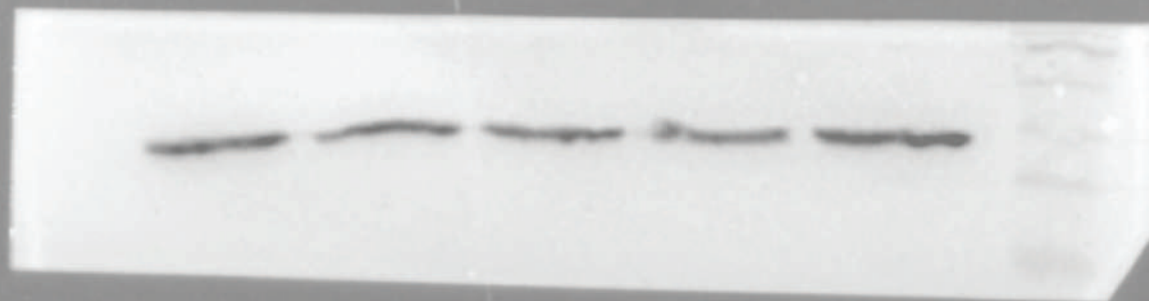

control LPS IFN-B VIT-D CO-Treat

Supplement: S1 File — (PDF) [file pone.0330865.s001.pdf]
